# History Repeating: Lessons From Citrus Fruits, Penicillin, and Delayed Adoption in Pediatric Cardiology

**DOI:** 10.1016/j.cjcpc.2026.02.005

**Published:** 2026-02-27

**Authors:** Colin J. McMahon, Conall Thomas Morgan

**Affiliations:** a Department of Paediatric Cardiology, Children’s Health Ireland at Crumlin, Dublin, Ireland; b Division of Pediatric Cardiology, The Hospital for Sick Children, Toronto, Ontario, Canada

**Keywords:** pediatric cardiology, adoption, innovation, lags, evidence-to-practice gaps

Pediatric cardiology has advanced remarkably in recent decades, reflecting progress in imaging, intervention, perioperative care, and long-term survival. Despite this, the movement of innovation from evidence to routine clinical practice remains inconsistent. Delayed diagnosis of critical congenital heart disease (CCHD) persists even in high-resource settings, despite validated strategies such as prenatal ultrasound and newborn pulse oximetry screening.^1^, ^2^, ^3^ Variability in the management of common congenital lesions further illustrates how uncertainty, heuristics, and institutional norms shape clinical decisions.^4^, ^5^, ^6^ Historical precedents, such as the prolonged delay in adopting citrus fruits to prevent scurvy and the slow dissemination of penicillin, highlight a recurring theme: evidence alone rarely drives practice change. Instead, structural inertia, cognitive bias, logistical constraints, and sociocultural resistance frequently impede progress. These dynamics remain visible across pediatric cardiology today.

## Citrus and the Long Road to Acceptance

In 1747, James Lind conducted one of the earliest controlled clinical trials, demonstrating the rapid recovery of sailors with scurvy treated with citrus fruits.^7^ His 1753 monograph, “A Treatise of the Scurvy,” clearly advocated for lemon and orange juice as preventive measures.^7^^,^^8^ Despite this, the British Admiralty did not mandate citrus rations until 1795, nearly 50 years later.^7^, ^8^, ^9^ Multiple forces contributed to this delay, including entrenched competing theories of disease causation, inconsistent preparation and storage of early citrus extracts, logistical challenges in naval provisioning, and institutional resistance to change. The citrus lag illustrates how cultural and systemic inertia can overwhelm even compelling clinical evidence.

## Penicillin and the Interwar Dissemination Gap

Penicillin’s trajectory parallels the citrus narrative. Although Fleming identified its antibacterial properties in 1928, the discovery lay largely dormant for more than a decade.^10^^,^^11^ Technical barriers to purification, limited funding, and a lack of commercial interest slowed progress. Only with the work of Florey, Chain, and colleagues, and the urgent clinical needs of World War II, did penicillin become available at scale, transforming infectious disease management.^10^^,^^11^ This pattern underscores a key principle: innovations require not only discovery but also supportive political, economic, and logistical ecosystems.

## Modern Echoes in Pediatric Cardiology

Persistent delays in translating innovation into practice remain evident in pediatric cardiology. One prominent example is newborn pulse oximetry screening for CCHD. Despite strong evidence demonstrating its benefit and endorsements from major professional bodies, global implementation remains inconsistent.^1^, ^2^, ^3^^,^^12^ Further examples include the adoption of percutaneous pulmonary valve implantation, hybrid procedures, and surgical planning using 3D models, which varies widely and often depends on local expertise, financial resources, and institutional culture.^13^^,^^14^ In low-resource regions, limited catheterization infrastructure, workforce shortages, and financial constraints contribute to profound inequities in access to these emerging innovations.^15^ Cognitive factors also play a role: clinicians frequently make decisions under conditions of uncertainty, where anchoring, framing, and availability bias can influence management choices.^4^, ^5^, ^6^

## Why Evidence-to-Practice Gaps Persist

Several forces conspire to result in persistent delays in implementing innovation in pediatric cardiology:

1. Cognitive bias: Status quo bias, ambiguity aversion, and memorable rare complications impede adoption.^4^, ^5^, ^6^
2. Evidence limitations: Many innovations rely on observational rather than randomized data.
3. Structural constraints: Screening programs and advanced procedures require trained personnel, equipment, and referral pathways.^12^
4. Professional culture: Innovations that disrupt established workflows face resistance.
5. Global inequities: Uneven expertise and resource allocation perpetuate disparities.^15^^,^^16^

## A Framework for Accelerating Adoption in Pediatric Cardiology

To reduce translation lag, a practical 5-part framework such as the following may be useful:

1. *Integrate implementation science into research design*: Trials in pediatric cardiology rarely include measures of feasibility, adoption, sustainability, or contextual barriers; these are core concepts in implementation frameworks such as Consolidated Framework for Implementation Research and Reach, Effectiveness, Adoption, Implementation, and Maintenance.
  o *Example:* A study of new fetal screening pathways could prospectively evaluate workflow compatibility, resource requirements, and stakeholder perceptions rather than only diagnostic accuracy.
  o *Result:* Research generates not only evidence of efficacy but also evidence for real-world uptake.
2. *Embed structured decision-making tools that surface bias:* Incorporating standardized pathways, checklists, and collaborative case reviews helps reduce cognitive heuristics that hinder adoption.
  o *Example:* A structured “CCHD escalation checklist” in neonatal units can reduce anchoring bias and prompt earlier referral to cardiology.
  o *Implementation link:* These tools function as behavioral interventions within complex clinical systems, improving reliability.
3. *Develop learning health systems using real-time data feedback:* Real-time dashboards showing local CCHD detection rates, catheterization outcomes, or access disparities can transform practice by providing immediate visibility.
  o *Example:* A congenital heart defect registry linked to electronic health records could flag delayed diagnoses in real time.
  o *Implementation link:* This aligns with learning health system principles such as continuous measurement, iterative improvement, and rapid-cycle evaluation.
4. *Strengthen global and regional partnerships to equalize access:* Effective models include twinning programs, virtual case conferences, shared training curricula, and pooled procurement of devices.
  o *Example:* A regional consortium sharing 3D-printing resources could accelerate adoption of advanced planning in centers without on-site capability.
  o *Implementation link:* This expands capacity-building, a key Consolidated Framework for Implementation Research domain.
5. *Reframe nonadoption as preventable harm*: Delayed uptake of proven interventions should be conceptualized similarly to failures in medication safety or infection control.
  o *Example:* Hospitals may integrate “time to CCHD diagnosis” as a quality metric, promoting accountability and improvement.
  o *Implementation link:* Using harm-based framing aligns with safety culture and motivates organizational change.

## Conclusion

The bitter history of citrus and penicillin teaches an uncomfortable truth. Generating evidence is only the first step. Without intentional strategies to support adoption, even life-saving innovations can stall for decades. Pediatric cardiology faces similar challenges today, from disparities in CCHD detection to uneven access to interventional technologies. By integrating implementation science, structured decision tools, learning health systems, collaborative partnerships, and a safety-driven reframing of nonadoption, the field can narrow its evidence-practice gap and avoid repeating the painful lessons of history.

## Funding Sources

The authors have no funding sources to declare.

## Disclosures

The authors have no conflicts of interest to disclose.
